# Temporal Evolution of Ischemic Lesions in Nonhuman Primates: A Diffusion and Perfusion MRI Study

**DOI:** 10.1371/journal.pone.0117290

**Published:** 2015-02-06

**Authors:** Xiaodong Zhang, Frank Tong, Chun-Xia Li, Yumei Yan, Doty Kempf, Govind Nair, Silun Wang, E. Chris Muly, Stuart Zola, Leonard Howell

**Affiliations:** 1 Yerkes National Primate Research Center, Emory University, Atlanta, Georgia 30329, United States of America; 2 Department of Radiology, School of Medicine, Emory University, Atlanta, Georgia 30322, United States of America; 3 Wallace H. Coulter Department of Biomedical Engineering, Emory University and Georgia Institute of Technology, Atlanta, Georgia 30322, United States of America; 4 Department of Psychiatry and Behavioral Sciences, School of Medicine, Emory University, Atlanta, Georgia 30322, United States of America; 5 Atlanta Veterans Affairs Medical Center, Decatur, Georgia 30033, United States of America; University of California San Francisco, UNITED STATES

## Abstract

**Background and Purpose:**

Diffusion-weighted imaging (DWI) and perfusion MRI were used to examine the spatiotemporal evolution of stroke lesions in adult macaques with ischemic occlusion.

**Methods:**

Permanent MCA occlusion was induced with silk sutures through an interventional approach via the femoral artery in adult rhesus monkeys (n = 8, 10–21 years old). The stroke lesions were examined with high-resolution DWI and perfusion MRI, and T2-weighted imaging (T2W) on a clinical 3T scanner at 1–6, 48, and 96 hours post occlusion and validated with H&E staining.

**Results:**

The stroke infarct evolved via a natural logarithmic pattern with the mean infarct growth rate = 1.38 ± 1.32 ml per logarithmic time scale (hours) (n = 7) in the hyperacute phase (1–6 hours). The mean infarct volume after 6 hours post occlusion was 3.6±2.8 ml (n = 7, by DWI) and increased to 3.9±2.9 ml (n = 5, by T2W) after 48 hours, and to 4.7±2.2ml (n = 3, by T2W) after 96 hours post occlusion. The infarct volumes predicted by the natural logarithmic function were correlated significantly with the T2W-derived lesion volumes (n = 5, r = 0.92, p = 0.01) at 48 hours post occlusion. The final infarct volumes derived from T2W were correlated significantly with those from H&E staining (r = 0.999, p < 0.0001, n = 4). In addition, the diffusion-perfusion mismatch was visible generally at 6 hours but nearly diminished at 48 hours post occlusion.

**Conclusion:**

The infarct evolution follows a natural logarithmic pattern in the hyperacute phase of stroke. The logarithmic pattern of evolution could last up to 48 hours after stroke onset and may be used to predict the infarct volume growth during the acute phase of ischemic stroke. The nonhuman primate model, MRI protocols, and post data processing strategy may provide an excellent platform for characterizing the evolution of acute stroke lesion in mechanistic studies and therapeutic interventions of stroke disease.

## Introduction

Diffusion-weighted imaging (DWI) and perfusion MRI are commonly used in the clinic to identify stroke infarction, estimate salvageable tissues after stroke insult, select an optimal therapeutic strategy, or examine treatment efficacy and predict the infarct growth [1–10]. Lesion development over time in stroke patients has been evaluated intensively with DWI and perfusion MRI [2], and this work has suggested that maximal lesion volume occurs in about 70 hours after stroke onset. In contrast, the maximal lesion volume is usually seen within 24 hours in rodent models of stroke [11–13], indicating a significant species difference in stroke evolution.

Old world nonhuman primates (NHPs) have brains that are gyrencephalic, i.e., structurally and functionally more similar to the human brain than in the rodent brain. NHP models allow for the examination of stroke evolution under conditions that can be well controlled, including the route of stroke insult, type of occlusion, lesion territory, and age of the subject. These variables are typically difficult to control in clinical studies of stroke patients. NHP models of stroke have been developed with middle cerebral artery (MCA) occlusion using a variety of approaches, including transcranial [14–16], transorbital [17–21], endovascular surgery via a neck incision [22–24]. These approaches vary in their difficulty and have other associated morbidity that can confound results. In comparison, an interventional approach via the femoral artery has been well established and routinely performed in neurointerventional procedures in the clinic. Previous results in macaque monkeys indicate it is minimally invasive, and most suitable for MRI evaluation of acute stroke [25,26].

The longitudinal evolution of stroke lesions in NHPs with permanent and transient occlusion has been studied previously for up to 30 days by Liu and colleagues in adult macaques [27], revealing the progressive growth of stroke infarct in the acute and chronic stage. However, the temporal evolution in the hyperacute stage is still poorly understood. In the present study, high resolution DWI and perfusion MRI measurements were used to further evaluate the spatial-temporal changes of stroke lesion after acute ischemic insult in a macaque model with permanent MCA occlusion.

### Methods and Materials

Adult rhesus monkeys (n = 8, female, 10–21 years old, 8.6±1.2 kg) served as subjects for the terminal study (n = 2) and survival study (n = 2 for 48 hours and n = 4 for 96 hours post stroke). As an essential aspect of our stroke model is the use of aged animals, female subjects were selected because there was a greater availability of aged female monkeys than male in our facility. All procedures were approved by the Institutional Animal Care and Use Committee (IACUC) at Emory University in a facility accredited by Association for Assessment and Accreditation of Laboratory Animal Care (AAALAC) and in compliance with the Animal Welfare Act and the Public Health Service Policy on Humane Care and Use of Laboratory Animals. The monkeys were housed in size appropriate cages and provided with perches, toys, and foraging devices as part of an environmental enrichment program. Prior to stroke studies, all monkeys were provided fresh water *ad libitum*, received nutritionally balanced primate biscuits (Monkey Diet Jumbo 5037, Lab Diet, St. Louis, MO) twice daily, and were supplemented with fresh produce on a rotating basis. The environment was maintained on a 12:12 h light:dark cycle at 60–75°F (22.6–23.9°C) and at a relative humidity of 30–70%. Between the time of the hyperacute phase and the 48-hour scan, subject RPF6 developed a small right basal ganglia and insular stroke. This lesion manifested seizure activity. Subject RJJ3 exhibited symptoms of left hemiparesis and was unable to maintain an upright posture without assistance. Therefore, these two subjects (RPF6 and RJJ3) were sacrificed after their 48-hour scan without recovery from anesthesia, in accordance with the approved IACUC protocol, due to their respective clinical condition and MRI findings at the 48 hour scan. The longitudinal MRI data acquisiton of one subject (PH1019) in the hyperacute phase was interrupted due to anesthesia sensitivity. Accordingly this subject was excluded in the temporal data analysis. Detailed information on subject characteristics is provided in Table 1.

**Table 1:** Demographic data of subjects.

Monkey ID	Age (years)	End Time Point (Hours post occlusion)	Body weight(kg)
RVI3	19.5	8	9.9
RCE3	20.0	8	7.2
RJJ3	21.5	48	8.2
RPF6	13.5	48	10.0
RFA5	18.0	96	9.3
RRI3	21.5	96	8.2
PH1019	13.5	96	9.2
RVG4	10.5	96	6.9
Mean±SD	17.2±4.2		8.6±1.2

### Stroke surgery procedure

The surgical procedure for the present study is as similarly reported previously [25,28]. Briefly, a micro-catheter was navigated to a small distal cerebral artery via the parent catheter to induce an infarction in the MCA territory in the right side. Once the catheter was in place, several separate pieces of 4–0 silk suture, measuring in various lengths (5–10 mm) were injected via syringe using normal saline to cause occlusion in angiographically selected MCA branches. The catheters were then removed from the femoral artery prior to MRI scanning. The entire stroke surgical procedure was approximately 1–1.5 hours in duration and included the time between the initial incision and the time to full occlusion occurred. Once the distal M2 section of MCA was occluded and confirmed with the c-arm fluoroscopy (SIREMOBIL Compact, SIEMENS Medical Solutions USA, Inc.), the animal was transported immediately to the MRI suite for imaging (taking ~30 minutes before starting the first MRI scan).

### Animal care in MRI and post stroke

The subject was initially anesthetized with 3–5mg/kg of telazol and then intubated and maintained using 1.0–1.5% isoflurane anesthesia for the duration of the MRI scan. A temperature-controlled recirculating warm water blanket was used to provide supplemental heat. The head of the subject was immobilized during MRI scanning with a custom-built head holder. All major physiological parameters were monitored and maintained within normal ranges [29]. The first two subjects (RVI3 and RCE3) were humanely euthanized immediately following the MRI scan without recovery from anesthesia. All other subjects were recovered from anesthesia after their first post-stroke scans.

After stroke surgery and MRI scans, each subject was housed individually without anesthesia and under continuous closed-circuit video recording. The neurological condition post stroke was assessed daily. Softened chow, supplementary food treats (approved fruit, vegetables and nuts) and juice (to aid in the evaluation of swallowing difficulty and mild facial paresis) were given with of the advice from the Yerkes veterinary department. In addition, water and food intake and urine and fecal output were monitored daily.

### MRI parameters

All images were acquired with a Siemens 3T clinical scanner (Siemens Medical Solutions USA, Inc, PA) and an 8-channel phase-array knee coil (Invivo Inc., FL). The imaging setting and protocols was similar as reported previously [28]. In the present study, anatomical structural images including MR angiograph (MRA), T2-weighted, FLAIR, DWI and perfusion MRI with gadolinium bolus injection (0.02mg/kg, Omniscan, GE Healthcare USA) were collected. MRA was acquired with the Time of Flight (TOF) protocol: FOV = 96mm × 96mm, slice thickness = 1mm, TR = 39ms, TE = 5.74ms, one slab, 40 slices per slab, data matrix = 448 × 448, single average; T2-Weighted images were acquired with fast spin-echo sequence with parameters: TR = 5000ms, TE = 115ms, FOV = 96mm × 96mm, data matrix = 256 × 256, slice thickness = 2mm, 2 averages; FLAIR was acquired with parameters: TR = 10000ms, TI = 2800ms, TE = 115ms, FOV = 96mm x 96mm, data matrix = 256 × 256, turbo factor = 17, slice thickness = 2 mm, 2 averages; DWI was acquired with the single-shot EPI sequence using conventional parallel imaging (GRAPPA[generalized autocalibrating partially parallel acquisition], R = 3) with TR = 5000ms/TE = 80ms, FOV = 96mm × 96mm, data matrix = 64 × 64, slice thickness = 1.5mm, 30 gradient directions with b-value = 1000s/cm^2^. Perfusion MRI for regional Cerebral Blood Flow (CBF) was derived with dynamic susceptibility contrast (DSC) MRI with gadolinium bolus injection and scanning parameters: TR = 2000ms/TE = 19ms, FOV = 96mm × 96mm, data matrix = 64 ×64, slice thickness = 1.5mm. Pre- and post-contrast T1-weighted structural images were conducted to evaluate the possible hemorrhage complication with T1-weighted magnetization-prepared gradient-echo (MP-RAGE) sequence with parameters: TR = 2500ms, TE = 3.33ms, FOV = 96mm × 96mm, flip angle = 8 degree, TI = 950ms, matrix = 192 × 192, slice thickness = 1mm, 112 slices, 1 average. DTI was repeated every hour in the Day 0 scan. Also, susceptibility-weighted imaging (SWI) was applied for detecting hemorrhage complication (data not shown). MRI was performed once at least one week before stroke surgery for screening purpose and collecting the baseline MRI data, and for up to 7 hours immediately after stroke surgery (Day 0) and rescanned at 48 hours (Day 2) and 96 hours (Day 4) hours post-occlusion.

### Histology

Subjects were euthanized without recovery from anesthesia after their last MRI scans by pentobarbital overdose and immediately intracardially perfused with saline followed by 10% buffered formalin according to well-established protocols approved by the IACUC of Emory University. The whole brains were removed and immersed in 10% buffered formalin. The brains were then blocked and sectioned at 50 μm using a freezing microtome. Selected sections were then stained with H&E, mounted and coverslipped.

### Data processing

DTI images were preprocessed, co-registered and averaged at each time point with the FSL software (www.fmrib.ox.ac.uk/fsl/). Lesion volumes from DWI were derived from the threshold (mean + 2 × standard deviation (SD)) of the DWI intensity on the contralateral side [30]. CBF maps were generated with the Siemens perfusion package in the scanner console. Lesion volumes from T2W images and CBF maps were delineated by comparing with the contralateral side using manual tracing (performed by CL, and further validated by SW (R = 0.98)). In particular, the hypoperfused regions were defined by referring to the CBF maps on the contralateral sides and their corresponding mean transit time (MTT) maps and DWI images to exclude the benign oligemia. The DWI infarct volumes were estimated with home-built Matlab scripts. H&E staining slides were digitized using a flatbed scanner for displaying and estimating the lesion volume of each stroke monkey with the imageJ software (www.NIH.gov) by manual tracing. Lesion volumes were calculated as the sum of the lesion region in each slice multiplied by the slice thickness.

A natural logarithmic function was used for fitting the temporal evolution of stroke infarct of each subject during the hyperacute phase. The equation used for curve fitting was:

$$V=C\times \ln(t)+V_{0}I$$

In which t is the time post occlusion (t > 0, unit: hour), V is the lesion volume (unit: ml) at the time t, C is the growth rate of the infarct volume per logarithmic time scale (hours), V_0_ is the baseline value (unit: ml) at the time t = 1 hour, ln is the natural logarithm.

The Spearman correlation analysis was conducted between the lesion volumes derived by DWI, perfusion, T2W, logarithmic function, and H&E.

## Results

Focal ischemic stroke with permanent occlusion was induced successfully in all subjects (n = 8) and confirmed with fluoroscopy before the subjects were moved for MRI. MCA occlusion was examined and validated with MRA in each subject for every MRI scan. The stroke lesion volume and location were further examined using DWI and perfusion MRI and validated with H&E staining (Table 2). Furthermore, the stroke territory in each animal was evaluated with T2-weighted and FLAIR images (not shown). No hemorrhagic transformation was observed in any subject. The infarction (shown in the last MRI scan of each animal) was observed in the frontal (7/8), parietal (3/8), and/or temporal (3/8) lobes, internal capsule (3/8), and striatum (3/8).

**Table 2:** Stroke lesion volumes measured with DWI, T2W, perfusion(CBF), and predicted with the natural logarithmic function (Unit: ml).

Monkey ID	6 hours post occlusion		48 hours post occlusion			96 hours post occlusion			Fitting parameters	
	DWI	CBF	CBF	T2W	Predicted	CBF	T2W	Predicted	Growth Rate	Baseline Volume
RVI3	3.3	7.4							1.04	1.03
RCE3	8.8	7.7							3.78	2.02
RJJ3	4.6	4.8	16.6	7.8	7.5				1.41	2.06
RPF6	0.59	1.36	1.73	1.08	0.97				0.21	0.17
RFA5	4.9	6.5	6.6	6.3	9.9	7.2	7.0	11.6	2.47	0.34
RRI3	1.6	3.2	1.9	2.5	2.5	2.1	2.5	2.8	0.43	0.86
RVG4	1.2	4.7	5.2	2.0	1.8	9.4	4.6	2.1	0.33	0.56
Mean±SD	3.6± 2.8	5.1± 2.3	6.4± 6.1	3.9± 2.9		6.4± 3.1	4.8± 1.9		1.38± 1.32	1.0± 0.76

### Stroke infarct volume evolution

**Fig 1:**
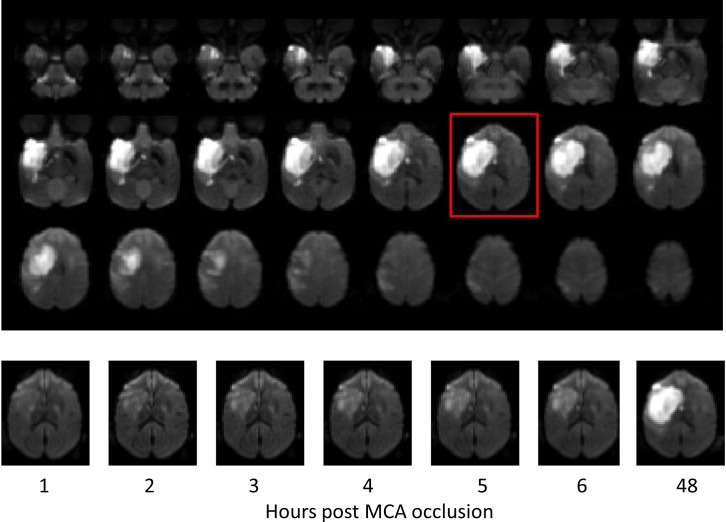
Stroke lesion evolution. Top: Axial DWI images of Subject RJJ3 at 48 hours post stroke show the entire infarct territory. Bottom: representative DWI images of RJJ3 demonstrate the infarct evolution at different time point (in hours) post MCA occlusion.

**Fig 2:**
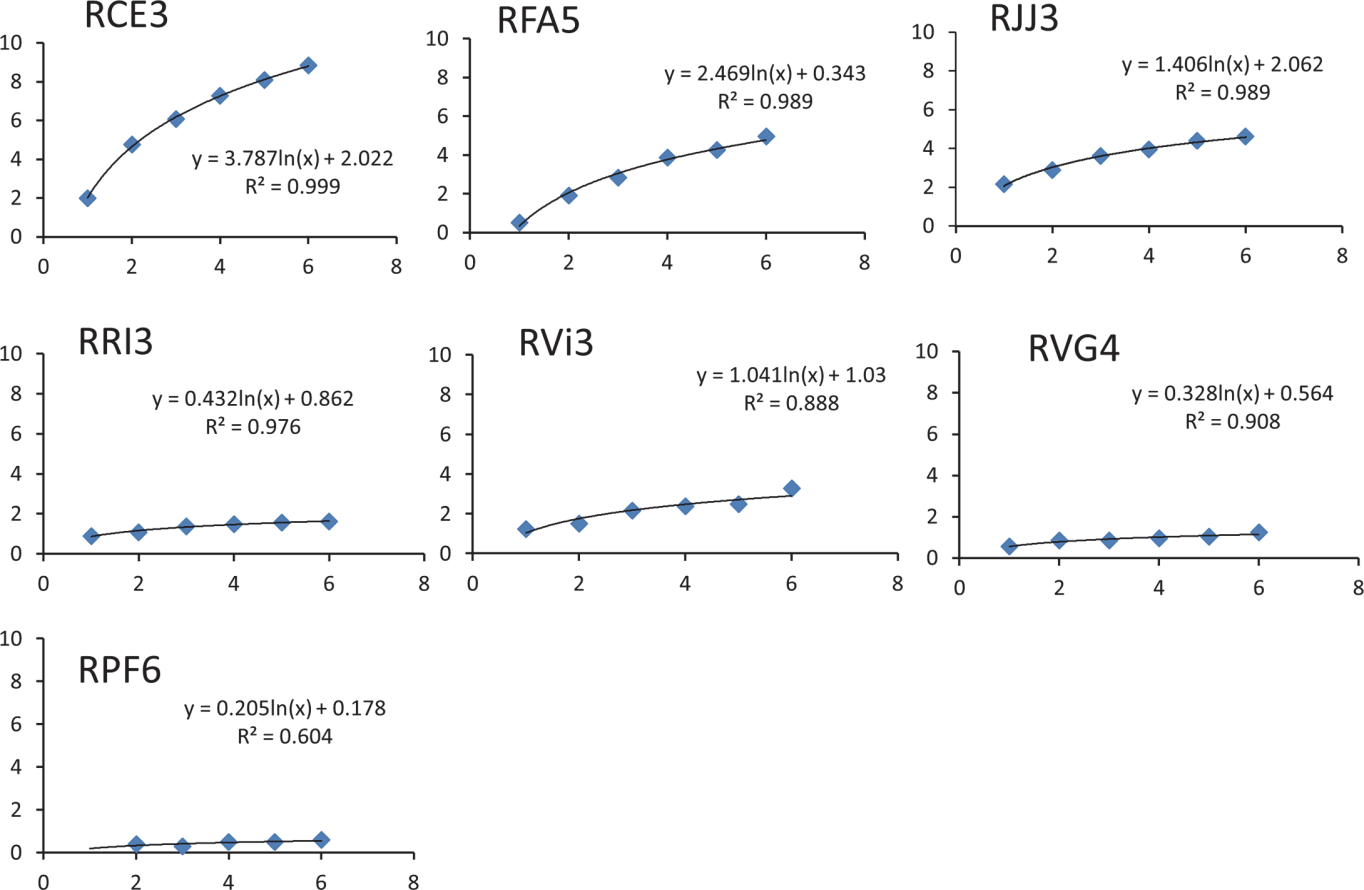
Temporal volumetric changes of stroke infarct in each subject during the hyperacute phase (1–6 hours). A natural logarithmic fitting was performed for each data set. Y-axis: volume (ml); X-axis: hours post MCA occlusion.

Stroke lesion was observed in every subject. The mean infarct volume is 3.57 ± 2.85 ml 6 hours post MCA occlusion (Table 2). The whole lesion volume at 48 hours and the progressive lesion changes of one representative slice during the entire study period are exhibited in Fig. 1. The temporal volumetric changes of each subject in the hyperacute phase (1–6 hours) are demonstrated and fitted perfectly with a natural logarithmic function (Fig. 2). The comparison of fitting the temporal evolution of averaged data indicates that the natural logarithmic function is better than a linear function even though the latter is also a reasonably good fit (R^2^ = 0.9949 vs R^2^ = 0.9638) (data not shown). Also, the exponential fitting was tested using the Origin software (OriginLab Corporation, Northampton, MA) and the result indicated that it did not work better than either the linear or logarithmic fitting (data not shown).

The natural logarithmic fitting of each subject shows that the stroke lesion develops typically in a logarithmic pattern in the hyperacute phase (Fig. 2). The corresponding mean growth rate is 1.38±1.32 ml per logarithmic time scale (n = 7). No linear or other correlated relationship was observed between growth rate and baseline lesion volume (the volume at 1 hour post MCA occlusion). The infarct volumes predicted by the natural logarithmic function are correlated significantly with the T2W-derived lesion volumes (n = 5, r = 0.92, p = 0.01) at 48 hours post occlusion but not at 96 hours (n = 3, r = 0.85, p = 0.20) likely due to the limited sample size. In comparison, the linear fitting gives a mean slope rate of 0.55±0.45 ml/hour in hyperacute phase, but fails to predict the lesion volumes at 48 hours post occlusion.

### CBF evolution in the lesion regions after ischemic occlusion

**Fig 3:**
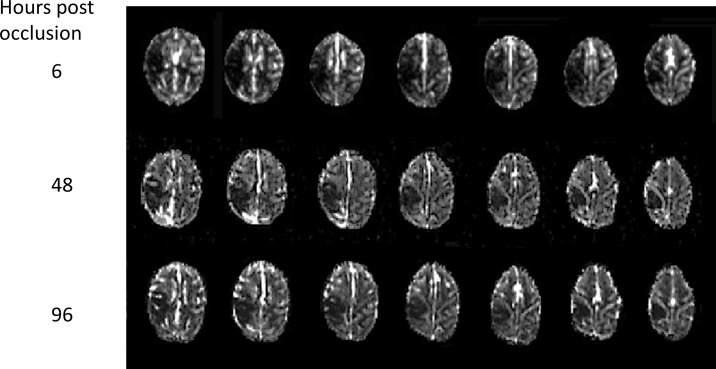
Serial axial CBF maps illustrate temporal perfusion changes in Subject RFA5 after permanent MCA occlusion.

Longitudinal changes of CBF in stroke-injured regions were examined using perfusion MRI with gadolinium bolus injection. Benign oligemia was seen in some subjects at 6 hours post occlusion by comparing with the MTT maps and/or corresponding final infarct territory, and excluded specifically in estimation of the hypoperfused volumes. Evolution of perfusion over time after stroke onset for a representative subject is shown (Fig. 3). The abnormal CBF volumes remained almost unchanged from the 6 to 48 hours post occlusion (5.1±2.3 ml vs 6.4±6.1 ml, n = 5), and 96 hours (6.4±3.1 ml, n = 3). When compared to volumetric changes illustrated in Table 2, the diffusion-perfusion mismatch still probably existed 6 hours post occlusion but diminished after 48 hours post occlusion.

**Fig 4:**
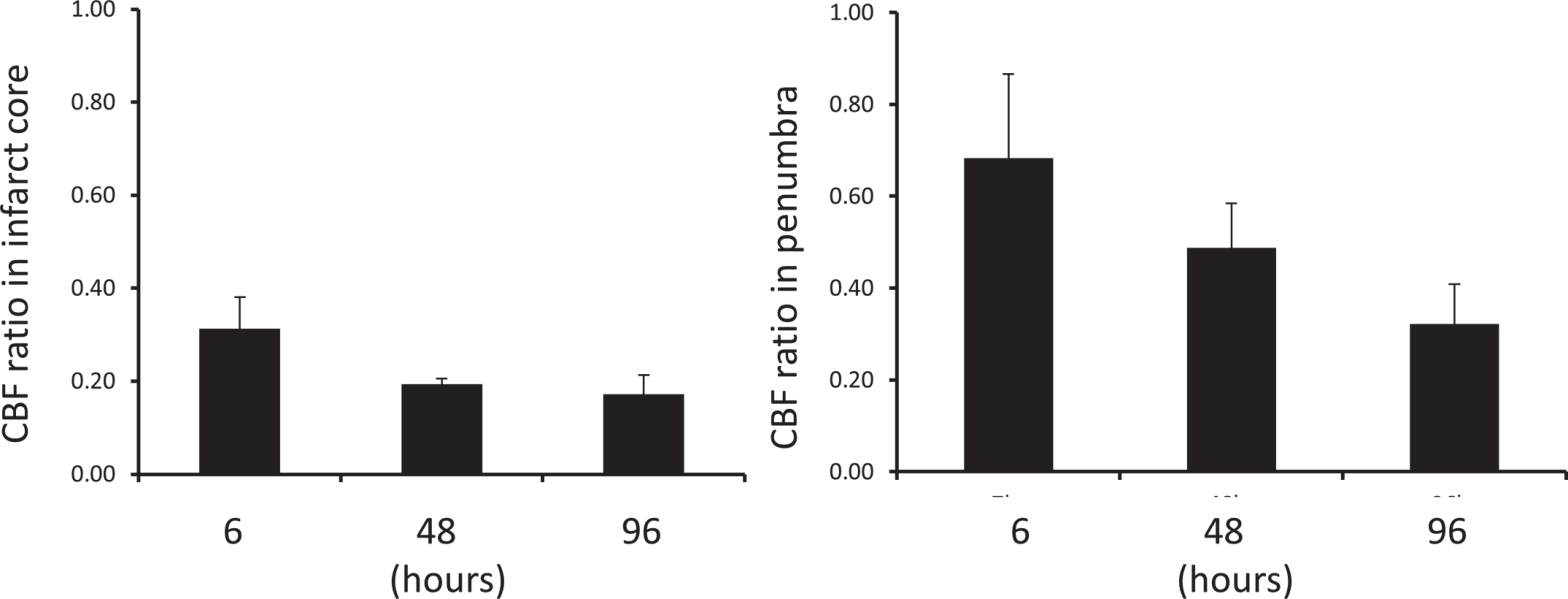
CBF ratio of ipsilateral/contralateral in infarction core (left) and penumbra (right) post MCA occlusion. Error bar: mean + Standard error.

The dynamic changes (n = 3) in CBF in the ischemic core and penumbra at 6, 48, 96 hours post occlusion are shown in Fig. 4, demonstrating specific developing patterns in the infarct and penumbra zones after acute stroke as expected. However, no significant difference was observed.

### Histology and correlation with MRI

**Fig 5:**
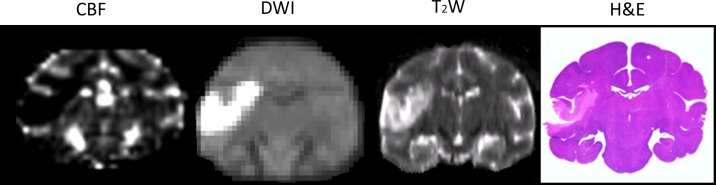
The stroke lesion is illustrated in the perfusion (CBF), diffusion (DWI), T2W images and H&E stained slice of the stroke monkey brain (RVG4).

**Fig 6:**
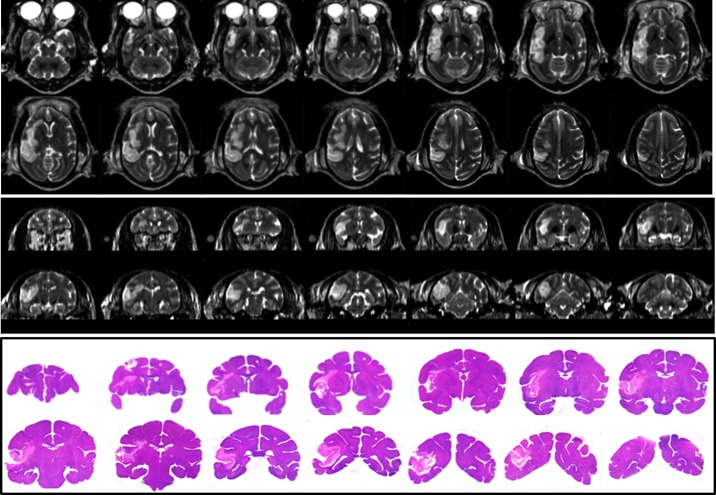
Illustration of ischemic lesions on T2W images and histology sections. Top: Axial T2W images of Subject RVG4. Middle: Reconstructed coronal T2W images of the same stroke monkey. Bottom: H&E staining of this stroke monkey brain sample.

H&E staining was performed on four brains. The T2W images and H&E stained slices from one stroke brain sample (RVG4) are demonstrated in Fig. 5 (with corresponding CBF map and DWI images and the whole brain slices in Fig. 6. The final infarct volumes derived from T2W (5.02 ± 2.18 ml, n = 4) are correlated significantly with their lesion volumes (3.85 ± 2.05ml, n = 4) from H&E staining (r = 0.999, p < 0.0001).

## Discussion

Permanent MCA occlusion was induced successfully in all eight adult macaque monkeys with more reproducible and appropriate cortical infarction than that observed in prior monkey studies using a similar procedure with minimally invasive surgery. The longitudinal DWI results demonstrate that the infarct volume evolves in a natural logarithmic pattern during the hyperacute phase, and the pattern can predict the typical lesion volume up to 48 hours post occlusion. Also, the perfusion results indicate that the diffusion-perfusion mismatch may last for more than 6 hours.

### Permanent MCA occlusion in NHPs

Permanent occlusion in the MCA vascular territory is generally employed to study the pathophysiological cascade after focal ischemia in preclinical studies [31]. Several kinds of emboli or occluders such as cyanoacrylate adhesive [32], nylon thread [23], silk sutures [33], sephacryl beads [34,35], and polystyrene spheres [22] have been used to induce permanent occlusion in the MCA territory in prior NHP studies of ischemic stroke. The interventional approach via the femoral artery is a neurointerventional procedure routinely performed in the clinic and minimally invasive. However, due to the technical challenge of the surgical procedure of MCA occlusion, the NHP models of stroke can be compromised with considerable variation in lesion infarcts [32]. In particular, severe lesions may result in high mortality in survival studies of NHPs. The present study demonstrates controlled stroke injury in adult macaques, indicating that the current surgical procedure with silk sutures is well suited to induce reproducible stroke in NHPs. Also, the resultant lesion volumes in this study were generally less than 10% of the total brain volume (an adult macaque brain volume = ~100 ml), indicating good survivability.

### Stroke infarct evolution

The temporal lesion volumetric changes in the hyperacute phase of permanent ischemic occlusion have been reported previously in rodent and macaque models of stroke [11,13,27]. However, the hourly changes of the stroke evolution during the entire hyperacute phase (1–6 hours) remains poorly understood in NHPs or other large animals or patients. In the present study with the NHP model, the temporal volumetric evolution after permanent occlusion was evaluated hourly for up to 6 hours with the high-resolution DWI.

The time-sensitive infarct volume and affected territory of each subject were well defined from the DWI images. Individual subjects showed different patterns of evolution due to initial lesion volume and territory differences in each subject, mimicking the stroke damage spectrum seen in the clinic. In particular, it was demonstrated that the stroke infarct evolved in a natural logarithmic pattern in the hyperacute phase of permanent ischemic occlusion, which could not be observed in a previous macaque study [27] due to the limited scanning time window in the hyperacute phase. Interestingly, the infarct volumes (n = 5) at 48 hours post occlusion are well predicted by the logarithmic function, indicating ischemic lesion may evolve in the logarithmic pattern over the entire acute stage. In contrast, the linear fitting also performed reasonably well in the hyperacute stage but obviously failed to predict the lesion volume in the sub-acute or later phase of ischemic occlusion as the maximal infarct volumes can be approximated in several hours in rodents or days in patients.

In addition, the growth rate and the corresponding baseline lesion volume (one hour post stroke) varies from subject to subject, indicating the growth rate of each infarction is probably independent of its initial lesion volume after stroke onset. Due to the limited sample size, such varieties cannot be fully evaluated in the present study. However, the subject-specific growth rates may be documented to reflect the wide spectrum of stroke lesions observed in preclinical and clinic studies.

It has been reported previously that the maximal infarct volume occurs at ~ 70 hours in stroke patients [2] while the stroke infarct evolution in rodents has been reported to stop growing after 3 hours of permanent MCA occlusion [11]. A previously cited macaque study by Liu and colleagues reported maximal infarction occurring at 24 hours post MCA occlusion [27]. By contrast, in the present study maximal infarct was approximated about 48 hours post occlusion. One explanation for the discrepancy between previous macaque studies and ours may be due to the lack of scans between 24 hour and 74 hours in Liu et al’s study. The results indicate the volumetric evolution in monkeys last much longer than that in the rodents but shorter than in stroke patients, suggesting that NHPs are more appropriate than rodents for modeling the stroke evolution observed in stroke patients.

### Abnormal CBF evolution

The diffusion-perfusion mismatch was still visible at 6 hours post occlusion but almost vanished in the 48 hours, suggesting that the mismatch may last more than 6 hours after stroke onset. But this finding will need to be validated with further histological examination. CBF in the infarct core changed very little after occlusion, as expected. In contrast, CBF in the penumbra (tissue at risk) declined gradually from 6 hours to 48 hours and 96 hours post stroke although no significant changes were observed in the group mean CBF. The results of individual subjects demonstrate the inter-subject difference in the hemodynamic changes after stroke and suggest that re-perfusion may still be effective to reduce stroke damage after hyperacute stage in some cases.

As perfusion-diffusion mismatch approximates the stroke penumbra, i.e., the salvageable brain tissue, it is recognized as the main target for developing therapeutic drugs in acute stroke and treatment evaluation [4,36,37]. Based upon the present macaque study, the mismatch was still observed at 6 hours post occlusion (p = 0.067). Observations of individual subjects indicated that the CBF evolution was quite variable probably due to the difference in lesion volume and territory and status of clotted vessel in each subject. In comparison with the mismatch findings in previous rat studies using permanent MCA occlusion [12,13], the diffusion-perfusion mismatch in NHPs lasted much longer than those (~3 hours) in rodents. In comparison, the mismatch in human stroke can exist more than 2 days [2,38]. The mismatch evolution differences across species suggest that the species difference may play a critical role in translational studies of stroke.

Abnormal changes in tissue diffusivity, sodium concentration, lesion volume, brain metabolites, pH-value, T2-value, et al, have been observed in many animal and/or human studies during acute stroke and are time-dependent [39–43]. To the best of our knowledge, no numerical model has been reported to delineate such temporal changes of quantitative MRI measures after stroke onset and can be possibly used for predicting lesion volume. The finding of the natural logarithmic pattern in permanent MCA occlusion suggests that such developing pattern may exist in other quantitative measures after stroke injury due to the association of lesion volume development with various quantitative measures for characterizing the infarction evolution. The numerical model for infarct prediction may be used in various preclinical stroke researches to reduce the sample size or the frequency of data acquisition post stroke and optimize drug administration and treatment plan in translational studies. In particular, the logarithmic pattern may be used to evaluate the efficacy of therapeutic interventions as each stroke subject’s predicted infarct volume can be used as his/her own reference. Certainly, its application and implication in stroke could be further explored in future studies by combining with multi-parameter MRI or other imaging techniques like CT or PET, and the potential application for clinical studies should be further investigated as well.

### Limitations and future research

This study reports the ischemic infarction evolves with a logarithm pattern in the acute phase in a macaque model with permanent MCA occlusion. Due to the formula’s intrinsic property, the pattern cannot be used to retroactively estimate the infarction near the t = 0 time point when the stroke lesion volume is assumed to be about zero, and also there are no imaging data available to reveal the ischemic infarct in such transient period immediately after stroke onset. In addition, the maximal infarct volume derived from the logarithm formula does not reach a plateau at the infinite point in time. This is not true in clinic. Therefore, the logarithm pattern cannot be used to predict the infarct volume in the chronic stage. Our results suggest the logarithm equation can be applicable to evaluate the temporal evolution up to 48 hours post stroke (and possible 96 hours post stroke if the sample size is sufficient to evaluate). More studies may be performed to delineate the evolution patterns beyond this specific period.

As macaque models mimic human stroke better than any other animal models, it is appropriate to use this excellent platform to explore the novel imaging techniques translational to clinical studies and evaluate the efficacy of neuroprotective treatments or other interventions in the future. In addition, unenhanced CT is widely used to identify the infarction in stroke diagnosis in the clinic. Such logarithm pattern may be also useful for modeling the infarct evolution in the diagnosis of acute ischemic stroke using CT.

## Conclusion

The temporal evolution of infarct volume during the hyperacute phase shows a natural logarithmic pattern in the adult macaque brains with permanent MCA occlusion. The logarithmic evolution of infarct volume could last up to 48 hours. The diffusion—perfusion mismatch still exists at 6 hours but almost diminishes at 48 hours post occlusion. The temporal evolution of stroke lesion in macaques largely resembles the findings seen in stroke patients, suggesting the NHPs, MRI protocols, and post data processing strategies can provide an excellent model for characterizing the evolution of stroke lesion and pathological stages for mechanistic studies and therapeutic interventions of stroke disease.
