# Correction: Temporal Evolution of Ischemic Lesions in Nonhuman Primates: A Diffusion and Perfusion MRI Study

**DOI:** 10.1371/journal.pone.0123613

**Published:** 2015-03-30

**Authors:** The PLOS ONE Staff

There is an error in the equation in the Data Processing subsection of the Methods and Materials section. The publisher apologizes for this error. Please view the complete, correct equation here:

$$\text{V}=\text{C x ln}\left(\text{t}\right)+{\text{V}}_{0}$$
